# Editorial: Advanced Learning

**DOI:** 10.3389/fpsyg.2021.712661

**Published:** 2021-06-29

**Authors:** Wilma Vialle, Heidrun Stoeger, Albert Ziegler

**Affiliations:** ^1^School of Psychology, University of Wollongong, Wollongong, NSW, Australia; ^2^School Research, School Development, and Evaluation, University of Regensburg, Regensburg, Germany; ^3^Educational Psychology and Research on Excellence, University of Erlangen Nuremberg, Erlangen, Germany

**Keywords:** deliberate practice, advanced learners, developmental trajectory, learning resources, giftedness

This issue on advanced learning focuses on the educational and developmental needs of advanced learners as they develop towards excellence. We speculated that those needs could be observed in at least three ways. The first is that the advanced learner requires educational interventions that are more closely aligned to the “deliberate practice” approach delineated by Ericsson et al. (1993). Ericsson et al. (1993) identified that the number of hours of deliberate practice differentiated among the performance levels of musicians. Deliberate practice can be described as individualised instruction whereby a teacher or coach identifies the goals and activities that need to be adopted by an individual during practice to improve their performance.

A second assumption is that advanced learners do not attain high levels of performance in the absence of environmental factors but the factors that support the talent developmental trajectory of advanced learners will not be the same as those that support them at earlier stages. The expertise reversal effect, for example, suggests that the instructional activities designed for novices may have a detrimental effect on more advanced learners Kalyuga (2007).

The third premise is the need for more tailored and well-designed learning resources to support talent development. Such learning resources include highly-specialised learning materials and curricula, expert teachers and coaches, mentors, and so on, which are purposefully designed to meet the individual's specific needs at a specific point in the talent development process. Again, this echoes the deliberate practice approach described earlier.

Ericsson's work on deliberate practice in expertise development is a key consideration, then, in this issue on advanced learning. Responding to critiques that questioned the extent of the contribution of deliberate practice to performance [see Macnamara et al. (2014)], in their paper Ericsson and Harwell carefully set out three criteria to determine whether practice regimens qualify as deliberate practice. The resulting analysis confirms the earlier work of Ericsson et al. (1993).

Gilar-Corbi et al. examined a range of non-cognitive variables to determine their impact on the academic outcomes of secondary students. Their comparison of underachieving and achieving gifted students revealed that the underachieving group had lower scores for learning strategies, goal orientations, self-concept, attitudes towards teachers, and perceptions of parent involvement in school. While these results underline the importance of particular personal and environmental resources in academic performance, what may be even more important is how gifted students may be assisted in learning to use the resources available to them more effectively. This does imply an approach that delivers the right supports to students at the right time for their developmental trajectory.

Barbier et al. compare achieving and underachieving gifted students. Their paper focuses on the lived experiences of the six participants who share intellectual gifted potential but differ in their achievement trajectories. The lived experience approach examines the individual within their environmental contexts. The authors demonstrate the role of supportive environments on the individuals' motivations, task engagement, and academic achievement.

Similar observation of the ways in which environmental supports or resources are reflected in individual psychosocial behaviours are evident in the contribution from Subotnik et al. These authors highlight the bi-directionality of the individual and environmental effects. They raise questions about how domains differ in terms of the nature of the interaction influence. It is important to note that talent development is not universal; domains play a part and thus, talent development in particular domains will require specific types of environment in which to flourish along with specific responses from individuals to those environmental influences.

Domain-specificity is also at the heart of the Reutlinger et al. study. In seeking to understand how individuals attain excellence in a specific domain, the authors examined the contribution of learning resources to talent development, and particularly whether domain-specificity of learning resources could be observed across two separate domains of school learning and learning a musical instrument. The study supported the domain-specificity of the resources, thereby reinforcing the conclusions of both the Ericsson and Harwell and Subotnik et al. papers that the talent development process requires specific attention that is unique to the individual's trajectory through a particular domain.

Zhou et al. questioned whether the filial piety correlates with the academic achievement of secondary students in China. Further, they investigated whether a similar pattern could be observed in other global settings. As hypothesised by the researchers, reciprocal filial piety was correlated with academic outcomes and with the need for autonomy. Their modelling also suggested that the association of the filial piety with academic achievement was via the need for autonomy. Analysis of global datasets, such as PISA, showed some evidence of similar patterns, thereby providing some support to the psychological construct's broader global influence. While additional research would be needed to further test these relationships, the findings do support other papers in this issue which speak to the critical role of familial learning resources in achievement outcomes.

While many of the papers in this issue focus on older children or adults, Howard and Vasseleu were interested in the early years, a critical period for the establishment of children's developmental trajectories. In this longitudinal study, the authors explored whether advanced preschoolers differed from their non-advanced peers in executive function and self-regulation. Their results confirmed that stronger cognitive development (reflected in combined executive functions and cognitive self-regulation) along with age and socioeconomic context consistently predicted stronger learning performances in the preschoolers. Interestingly, though, the advanced learners attained lower behavioural self-regulation ratings, which the authors speculate may promote rather than constrain learning.

The importance of mentors in supporting the development of advanced learners has been well-established but has predominantly focused on the mentor-mentee relationship. What is missing from these analyses is the role that peers play in the outcomes arising from mentoring. In their study of female secondary school students participating in an online STEM mentoring program, Hopp et al. addressed this gap in the literature. The longitudinal study showed that the measured outcomes were indeed influenced by the mentees' peers in the program but this effect was moderated by age, whereby younger mentees became more similar and older mentees became more dissimilar. There was also some evidence of the size of the peer groups bearing some influence for the younger mentees. This interesting research, in demonstrating age-related differences, reinforces the theme demonstrated elsewhere in this issue that the development of advanced learners requires the delivery of the right educational experiences at the right time for the individual in their trajectory towards outstanding achievement.

## Author Contributions

WV drafted the editorial. HS contributed to the design of the issue and to the writing of the editorial. AZ conceptualised the design of the issue and reviewed the draft of the editorial. All authors contributed to the article and approved the submitted version.

## Conflict of Interest

The authors declare that the research was conducted in the absence of any commercial or financial relationships that could be construed as a potential conflict of interest.
